# A New PKD-1 Mutation Discovered in a Black African Woman With Autosomal Polycystic Kidney Disease

**DOI:** 10.5812/numonthly.6651

**Published:** 2013-03-30

**Authors:** Sidy Mohamed Seck, Serigne Guèye, Boucar Diouf

**Affiliations:** 1 Internal Medicine and Nephrology Department, Faculty of Health Sciences, University Gaston Berger, Saint-Louis, Senegal; 2 Nephrology Department, University Hospital Aristide Le Dantec, Dakar, Senegal

**Keywords:** Polycystic Kidney, Autosomal Dominant, PKD-1, Mutation, African Continental Ancestry Group

## Abstract

Autosomal polycystic kidney disease (ADPKD) is a genetic disorder with two causal PKD-1 and PKD-2. Genetic studies have demonstrated an important allelic variability between patients but few data are known about genetic variants in African populations. We report a new mutation found in a 41-year old women with mild chronic kidney disease secondary to ADPKD. Molecular genetic testing found a deletion of 2 nucleotides A and C at positions 7290 and 7291 followed by insertion of a 5-base pair (CTGCA) located in exon 18 of the PKD1 gene. This newly identified frame shifting was compared to the PKD gene database but no similar mutation was yet reported. Other screened family members did not present any mutation.

## 1. Introduction

Autosomal dominant polycystic kidney disease (ADPKD) is the most frequent cause of hereditary nephropathy with two identified causal genes, PKD1 and PKD2 located respectively on chromosome 16 and chromosome 4 (1). These genes encode cell membrane proteins called polycystin-1 (PC1) that interacts with polycystin-2 (PC2) to function as cell surface signaling receptor and as a mechano-sensor in renal primary cilia involved in renal tubular differentiation pathways (2).

At the clinical level, ADPKD is generally a late-onset multisystem disorder characterized by development of cysts in kidneys and often in other organs like liver, seminal vesicles, pancreas, and arachnoids membrane (1). Approximately 50% of individuals with ADPKD have end-stage renal disease (ESRD) by age 60 years.

A variety of mutations with different clinical significance has been reported mostly in white and African American patients (3). However, despite a growing literature on epidemiological and clinical patterns of ADPKD, data on genetic variation in black African populations are scarce (4).

We report a case of new mutation identified in a black woman with ADPKD.

## 2. Case History

A 41-year old woman presented at nephrology outpatient clinic with resistant hypertension, chronic flank pain and a past episodes of macroscopic hematuria. Her mother died 10 years ago from hypertension and kidney cysts. She had two sisters aged 36 and 30 years old respectively. Physical examination found two abdominal masses at palpation of lateral quadrants. Serum creatinine level was 1.34 mg/dL with an estimated glomerular filtration rate (GFR) of 58 mL/min/1.73 m^2^. Blood urea nitrogen, serum hemoglobin and urinalysis were normal. Renal ultrasound examination showed bilateral enlarged kidneys with multiple cysts of variable sizes. There was no extra-renal cyst localization and echocardiography was normal. The diagnosis of ADPKD was evoked and a genetic conformation was asked.

Molecular genetic testing used a sequence analysis method targeting commonly reported exons for PKD1 mutations (3). A 5 mL peripheral venous blood sample was taken from the patient for analysis. Two samples of blood were collected from age-matched controls without any kidney disease. DNA was extracted from peripheral blood (PB) cells by standard procedure. Exons were PCR amplified using the primers constructed from the consensus PKD1 gene sequence according to standard protocols (3). Complete PCR products (5 μL) were mixed with 5 μL 50 mM NaOH and 95% formamide and electrophoresed in 6% PAGE 1 × TBE gel with 2.5% crosslinking at 250 V for 16 h at 4°C. The gel was then stained with 0.5 mg/m/ethidium bromide and photographed under UV light. DNA samples exhibiting shifted bands were amplified and sequenced using ABI Prism™ 310 Genetic Analyzer.

**Figure 1.**
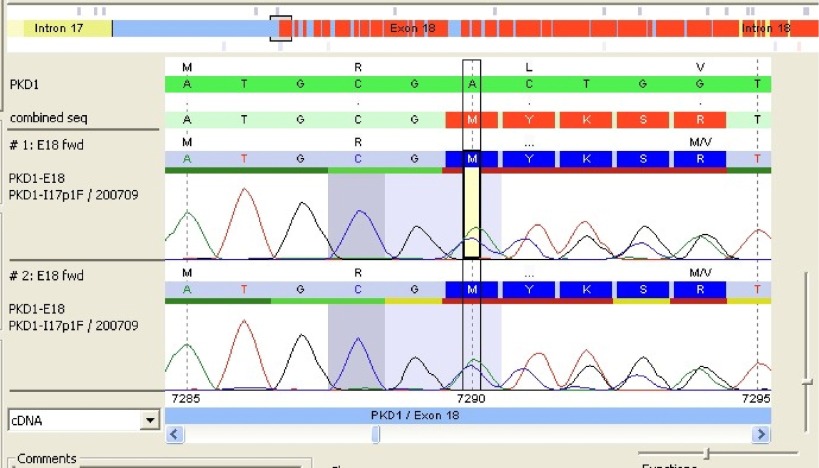
The Result of Genetic Sequence Showing the Frameshifting in Exon 18 of Patient’s PKD-1 Gene The 3 coloured horizontal bands represent respectively the reference sequence (green band), the sequence found in patient’s gene (blue band) and the combination of these 2 sequences (red band).

**Figure 2.**
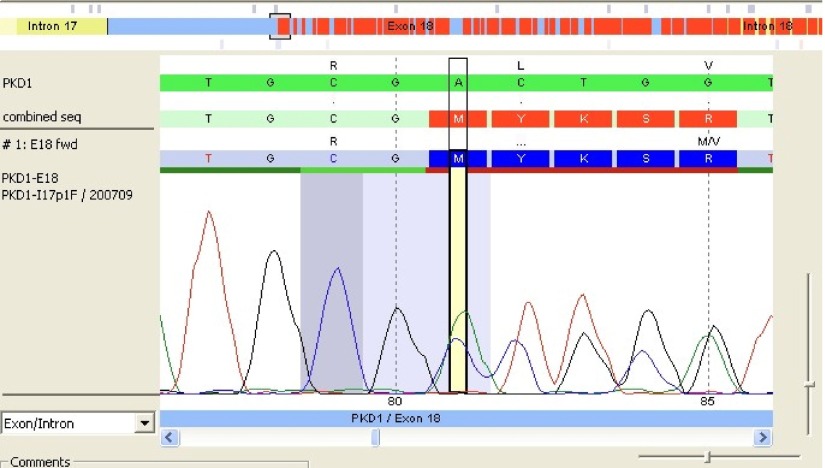
The Patient’s Genetic Sequence in Exon 18With a Double Pic Confirming That Mutation Was Heterozygote

After comparison with PKD1 reference sequence (RefSeq), our patient's PKD1 gene showed a deletion of 2 nucleotides A and C at positions 7290 and 7291 followed by insertion of a 5-base pair (CTGCA). This frameshift mutation was located in exon 18 of PKD1 RefSeq sequence (Figure 1). The analysis also revealed the presence of a double pic at this position showing that the mutation was heterozygote. The query sequence (c.7290_7291 delins CTGCA) was then compared to the PKD gene database but no similar mutation was yet reported (Figure 2).

Management of patient included prescription of antihypertensive drugs (ACE inhibitors), dietary advice and genetic counselling. The other family members performed ultrasound examination and multiple bilateral kidney cysts were present in one sister. Her molecular genetic testing did not find any mutation.

## 3. Discussion

ADPKD is a genetic disease characterized by an important allelic variability with many variants described (1, 3). The present study is the first description of the PKD1 variant in ADPKD patients living sub-Saharan Africa. We identified a new genetic variant of PKD1 in an adult woman with mild decrease of GFR secondary to ADPKD.

The most frequently reported types of mutations with pathogenic significance are deletions, insertions, splices, frameshifts, substitutions and nonsenses (3). In 85-90% of patients mutations concern PKD1 gene and PKD2 in 10-15% of cases (1). We found a new mutation located in the exon 18 of the PKD1 gene. Molecular genetic analysis is rarely reported in many African countries because of unavailability (4, 5).

Moreover, genetic testing is not always mandatory in patients with ADPKD. The diagnosis relies primarily on imaging studies of the kidneys (ultrasound, CT scan, magnetic resonance imaging) and clinical history. Genetic analysis is recommended in cases were familial history and kidney imaging findings are equivocal (1). In this patient aged 40-59 years with a PKD history in her mother, the presence of more than two cysts in each kidney was very suggestive of ADPKD according recent unified criteria (6). However genetic evaluation was performed to have an idea of causal mutation. Method of genotype analysis is variable according to clinical context and familial history.

In this patient with a family history of ADPKD, direct sequencing based on data derived from previously identified mutations of PKD1 is the recommended method for molecular genetic testing and its sensitivity is estimated to 92% (7). Testing by linkage analysis is possible in larger families using highly informative microsatellite markers flanking PKD1 and PKD2 but this method relies on accurate clinical diagnosis of ADPKD among family members (7).

The overall detection rate for ADPKD genetic screening is approximately 88% (8, 9) and approximately 10% of individuals who undergo comprehensive genetic screening of PKD1 and PKD2, no mutation is identified (7, 9). It is unclear if such cases are results of missed mutations at the known loci or further genetic heterogeneity.

In fact, about 70% of PKD1 mutations are unique and 25% of its variations are missense. Thus, pathogenicity of some allelic variant is difficult to prove. However, the development of specific algorithms to score missense variants can help to assess pathogenicity likelihood (7, 8). In our patient it is highly probable that this newly identified frameshifting is a pathogenic mutation.

More recently a new 6-base pair deletion in the middle of the exon 40 sequence was reported in Indian patients (10). Familial cases of ADPKD have been described in West African patients but genetic analysis was not available (5). It is probable that our patient's mutation might be present in her family but further genetic testing of other family members is required. Also, utility of identifying a new mutation in a single patient is limited in terms of genetic counselling and outcome prediction. Nevertheless, it is a first step to future multicentre studies for better description of genetic variants in African ADPKD patients.
